# Multicomponent meningococcal B vaccination (4CMenB) of adolescents and college students in the United States

**DOI:** 10.1177/2051013616681365

**Published:** 2017-01-06

**Authors:** Angelika Banzhoff

**Affiliations:** GSK, Emil-von-Behring-Strasse 76, 35041 Marburg, Germany

**Keywords:** adolescent vaccination, meningococcal diseases, meningococcal infections, meningococcal vaccines, *Neisseria meningitidis* serogroup B

## Abstract

Meningococcal disease is rare, easily misdiagnosed, and potentially deadly. Diagnosis in the early stages is difficult and the disease often progresses extremely rapidly. In North America, the incidence of invasive meningococcal disease (IMD) is highest in infants and young children, with a secondary peak in adolescents, a population predominantly responsible for the carriage of disease. *Neisseria meningitidis* serogroup B (MenB) accounts for a large proportion of meningococcal disease in North America, with documented outbreaks in three universities in the United States (US) during 2008–2013. Vaccination is the most effective way to protect against this aggressive disease that has a narrow timeframe for diagnosis and treatment. 4CMenB is a multi-component vaccine against MenB which contains four antigenic components. We describe in detail the immunogenicity and safety profile of 4CMenB based on results from four clinical trials; the use of 4CMenB to control MenB outbreaks involving vaccination at two US colleges during outbreaks in 2013–2014; and the use of 4CMenB in a Canadian mass vaccination campaign to control the spread of MenB disease. We discuss the reasons why adolescents should be vaccinated against MenB, by examining both the peak in disease incidence and carriage. We consider whether herd protection may be attained for MenB, by discussing published models and comparing with meningitis C (MenC) vaccines. In conclusion, MenB vaccines are now available in the US for people aged 10–25 years, representing an important opportunity to reduce the incidence of IMD in the country across the whole population, and more locally to combat MenB outbreaks.

## Introduction

### Invasive meningococcal disease

Meningococcal disease is a rare infection, with incidence of around 0.7 per 100,000 persons in Europe [European Centre for Disease Prevention and Control, 2012] and 0.14 per 100,000 persons in the United States (US) [Centers for Disease Control and Prevention, 2014]. It is potentially a deadly disease, with case-fatality rates of 8–14%, with 10–20% of survivors having sequelae such as limb amputation, hearing loss and neurological complications [Pace and Pollard, 2012; WHO, 2015; Harrison, 2015; Thompson *et al.* 2006]. Diagnosis in the early stages is difficult and it is easily misdiagnosed, partly as community physicians see few cases during their careers and partly because early symptoms are similar to that of other diseases; classical features associated with invasive meningococcal diseases (IMDs), such as hemorrhagic rash and meningism, appear relatively late during the development of illness resulting in a delay in diagnosis [Rosenstein *et al.* 2001; Thompson *et al.* 2006]. Furthermore, the disease often progresses extremely rapidly and patients may be close to death by 24 h [Thompson *et al.* 2006; WHO, 2015].

Meningococcal disease is caused by *Neisseria meningitidis*, a Gram-negative, aerobic diplococcus. Different strains are classified into serogroups according to the immunogenicity of their capsular polysaccharides. There are 12 serogroups and most cases of meningococcal disease globally are caused by six of them: A, B, C, W, X and Y; of these, serogroups B, C and Y are common in North America [WHO, 2015; Harrison, 2010]. Initial colonization of the pharynx by *N. meningitidis* normally leads to asymptomatic carriage. In the vast majority of carriers, colonization does not develop to invasive disease, which is a relatively rare occurrence [Christensen *et al.* 2010].

In North America, incidence of IMD is highest in infants and young children, with a secondary peak in adolescents [Baccarini *et al.* 2013]. However, meningococcal carriage occurs predominantly in adolescents; prevalence increases through childhood from 4.5% in infants to a peak of 23.7% in 19-year olds, then decreases in adulthood [Christensen *et al.* 2010]. Carriage is an important risk factor for meningococcal diseases, which has been previously associated with social behavior in young individuals [MacLennan *et al.* 2006; Harrison *et al.* 2008]. A recent meningococcal carriage study in Rhode Island, USA, undertaken in response to an outbreak on a college campus, reported around 25% carriage in college students (176/717 students); 18% (132/717) carried nongroupable *N. meningitidis* and 4% (31/717) carried serogroup B [Soeters *et al.* 2015]. *N. meningitidis* serogroup B (MenB) accounts for a large proportion of meningococcal disease in North America [Racloz and Luiz, 2010]. In the US, more than 60% of cases among children younger than 5 years and 40% of cases among the entire population are caused by MenB [Centers for Disease Control and Prevention, 2014]. This serotype also accounts for around a third of cases of IMD [Halperin *et al.* 2012; O’Ryan *et al.* 2014]. Infection is generally endemic, but outbreaks can occur unpredictably, particularly in high-risk populations such as persons living in close quarters and military personnel [Rosenstein *et al.* 2001]. An outbreak at a US university during 2008–2010 resulted in 13 identified cases (of which 10 were confirmed to be caused by MenB), with 1 death [Mandal *et al.* 2013]. In 2013, two unrelated university campuses (Princeton University and University of California, Santa Barbara, USA) experienced outbreaks with a combined 13 cases and 1 death [Centers for Disease Control and Prevention, 2015; McNamara *et al.* 2015]. In 2015, an outbreak in Oregon reported six cases (of which one was fatal); in an outbreak in Rhode Island in February, two cases of MenB were reported [Harrison, 2015; Soeters *et al.* 2015].

Prevention through vaccination is the best defense against an aggressive disease that leaves little time for intervention, has a high case-fatality rate in spite of antibiotic treatment and may lead to major sequelae [Rosenstein *et al.* 2001; Thompson *et al.* 2006; Centers for Disease Control and Prevention, 2016].

For IMD, quadrivalent polysaccharide or polysaccharide-protein conjugate vaccines are available for protection against meningococcal serogroups A, C, W and Y. Polysaccharide vaccines have been used for decades and are well tolerated. However, they are poorly immunogenic in infants and lack substantial herd protection [Harrison, 2006, 2015]. Conjugate vaccines, where the bacterial polysaccharide is covalently bound to a carrier protein, show higher vaccine effectiveness and immunogenicity in infants; they also offer substantial herd protection and are now widely used in immunization programs [Harrison, 2006, 2015].

In the US, the Advisory Committee on Immunization Practices (ACIP) has recommended since 2005, the routine vaccination against serogroups A, C, W and Y for individuals 11–18 years of age [Centers for Disease Control and Prevention, 2013; Harrison, 2015].

There were no MenB vaccines available until 2014. There are now two vaccines that are marketed and licensed under the accelerated approval of biological products regulations for use in individuals aged 10–25 years [US Food & Drug Administration, 2014a, 2015b]. This approval is granted with the caveat that postmarketing clinical studies should be performed in order to further investigate the immunogenicity and tolerability of the product, to assess its effectiveness in the targeted population in the US, and to verify and describe its clinical benefit in relation to the ultimate outcome. The approval may be withdrawn or modified if the studies fail to demonstrate clinical benefit or to meet any US Food & Drug Administration (FDA) requirements.

In 2015, ACIP issued a category B recommendation to vaccinate against MenB in individuals aged 16–23 years to provide short-term protection against most strains of serogroup B meningococcal disease, with a preferred vaccination age of 16–18 years. Category B recommendations allow individual physicians to make clinical decisions and to weigh the benefit *versus* the risk. Vaccination is also recommended for certain individuals aged ⩾10 years who are at increased risk for serogroup B disease: persons with persistent deficiencies in the complement pathway, with functional or anatomic asplenia, microbiologists working routinely with *N. meningitidis* isolates, and persons likely to be exposed during outbreaks of MenB meningococcal disease [Folaranmi *et al.* 2015]. The two MenB vaccines are not interchangeable, so the vaccination series must be completed with the same vaccine [MacNeil *et al.* 2015].

The rLP2086 (TRUMENBA™, Pfizer, Phila-delphia, USA) vaccine consists of two variants of factor H-binding protein (fHbp), one from subfamily A and one from subfamily B [US FDA, 2014a]. It was licensed in the US in October 2014 for use in individuals 10–25 years of age, with a three dose schedule administered over a 6-month period [US FDA, 2014b]. Recently, a two-dose schedule with the two doses administered 6 months apart has been licensed. The safety of rLP2086 was evaluated in seven clinical trials, of which four were randomized controlled and three were supportive, noncontrolled trials. Immunogenicity and safety of rLP2086 has been reviewed elsewhere [Shirley and Dhillon, 2015].

This paper is an overview of the immunogenicity and safety of the 4CMenB vaccine (BEXSERO^*™*^, GSK Vaccines, Siena, Italy) in adolescents and young people, the age category targeted for short-term immunization against MenB-caused diseases in the US.

### 4CMenB

4CMenB is a multi-component MenB vaccine containing four antigens [O’Ryan *et al.* 2014]. The development of 4CMenB was built on the successful use of strain-specific Outer Membrane Vesicle (OMV)-based vaccines, used in New Zealand, Cuba and Norway, which demonstrated the effectiveness of protein-based vaccines against MenB [Holst *et al.* 2013]. 4CMenB is one of the first MenB vaccines that may be effective against multiple MenB strains, dependent on its antigenic components: factor H-binding protein (fHbp) from the B sub-family, *Neisseria* adhesin A (NadA), *Neisseria* heparin binding antigen (NHBA), and porin A (PorA) P1.4 [O’Ryan *et al.* 2014]. Each component has been carefully selected to target an improved strain coverage, a potentially high protection against mutations, and synergistic killing: fHbp binds factor H, thus reducing bacterial survival in the blood [Madico *et al.* 2006; Schneider *et al.* 2009], NadA is important for colonization and promotes adherence to and permeability of epithelial cells [Comanducci *et al.* 2002; Capecchi *et al.* 2005; Mazzon *et al.* 2007]. NHBA is conserved in practically all *Neisseria* strains and is involved in reducing bacterial survival in the blood [Bambini *et al.* 2009; Serruto *et al.* 2010], while PorA is an OMV protein from the New Zealand outbreak strain NZ98/254 shown to induce strain-specific bactericidal response [Martin *et al.* 2006]. Recombinant antigens from bacterial surface proteins fHbp and NHBA are prepared as fusion proteins with two antigenic carrier proteins, and NadA is used as a single protein [O’Ryan *et al.* 2014]. A tool developed to assess the distribution of MenB strains likely to be covered by the 4CMenB vaccine, the Meningococcal Antigen Typing System (MATS), has estimated that the combination of these four components might cover over 90% of MenB strains in the US [Kim *et al.* 2012]. This is the highest 4CMenB coverage predicted by MATS anywhere in the world [O’Ryan *et al.* 2014; Medini *et al.* 2015], but is yet to be confirmed by post-vaccination surveillance data. Moreover, as the genes encoding for fHbp, NadA and NHBA can be expressed in other meningococcal serotypes, recent data seem to suggest that the protection offered by vaccination with 4CMenB might extend to non-B strains as well [Bianchi *et al.* 2015].

4CMenB was first approved in the EU in 2013 and is now approved in over 35 countries, including the US, Canada, Europe, Australia, Chile, Argentina and Brazil [Novartis, 2015]. 4CMenB, administered with a two-dose schedule, was approved for use in the US by the FDA in January 2015 for individuals 10–25 years of age, for active immunization to prevent invasive MenB disease, according to a flexible two-dose schedule in which the second dose can be administered 1–6 months after the first dose [US FDA, 2015a, 2015b].

Between 2013–2014, prior to 4CMenB approval in the US, MenB outbreaks occurred on two US college campuses. In response, the US FDA authorized the use of 4CMenB as an investigational vaccine at these universities under a Centers for Disease Control and Prevention (CDC)-sponsored Investigational New Drug application [Patel *et al.* 2014; McNamara *et al.* 2015]. Over 15,000 individuals were vaccinated and over 28,000 doses of 4CMenB were administered during vaccination campaigns between December 2013 and May 2014.

## Immunogenicity in adolescents and young adults based on clinical trials

As part of the clinical development program, immunogenicity of 4CMenB in adolescents was studied in four clinical studies V72_41, V72_29, V102_03 and V72P10 [Perrett *et al.* 2015; Read *et al.* 2014; Block *et al.* 2015; Santolaya *et al.* 2012]. Human complement serum bactericidal activity (hSBA) was assessed against three indicator strains chosen to determine the immunogenicity of individual vaccine components in studies V72_41 and V72P10: strain 44/76-SL for fHbp, strain 5/99 for NadA, and strain NZ98/254 for PorA P1.4. Responses to NHBA were initially assessed by enzyme-linked immunosorbent assay as no suitable indicator strain had been identified at the time of these clinical trials and these assessments were evaluated for registration of the 4CMenB vaccine in the US. In study V102_03, strain M14459 for fHbp, strain M01-0240364 for NadA, strain NZ98/254 for PorA and strain M07-0241084 for NHBA were used, but immunogenicity results from this study were not analyzed for US registration.

The main immunogenicity endpoints in the four 4CMenB clinical trials were different. The hSBA titers of ⩾1:4 were used in studies V72_29 and V72P10. Assessments were performed by the Public Health England laboratory in Manchester, UK (V72_29) and a GlaxoSmithKline laboratory in Marburg, Germany (V72P10). The ⩾1:4 cut-off was initially selected based on Goldschneider’s data from the 1960s which showed a correlation between bactericidal titers of ⩾1:4 and protection from meningococcal disease [Goldschneider *et al.* 1969]. However, at a later stage of the clinical development plan, a more stringent criterion of ⩾1:5 was employed to ensure with 95% confidence that the titer achieved is at least 1:4. As a consequence, in studies V72_41 and V102_03, hSBA titers of ⩾1:5 were used as the main endpoints (evaluated by the same GlaxoSmithKline laboratory) [European Medicines Agency, 2015; US FDA, 2015b]. These two immunogenicity endpoints were used for registrations outside of the US [European Medicines Agency, 2015].

In the US, two other immunogenicity endpoints were used for registration: the proportion of patients who achieved a ⩾4-fold increase in hSBA titers 1 month after the second dose of 4CMenB, and a composite response [US FDA, 2015b]. Both of these endpoints were *post hoc* and unplanned analyses. For the analyses, the lower limit of quantitation (LLOQ) was defined by the US FDA according to each antigen and laboratory. The ⩾4-fold increase was defined as: a post-vaccination hSBA titer ⩾1:16 for participants with pre-vaccination titers <1:4, a post-vaccination titer at least 4-fold of the LLOQ for participants with pre-vaccination titers ⩾1:4 but <LLOQ, and a post-vaccination 4-fold rise for participants with pre-vaccination titers ⩾LLOQ. The composite response endpoint was defined as hSBA titers ⩾LLOQ for all three indicator strains.

V72_41 was a phase III, randomized, observer-blind study conducted in Australia and Canada which enrolled adolescents 11–17 years of age [ClinicalTrials.gov identifier: NCT01423084] [Perrett *et al.* 2015]. A total of 344 adolescents were enrolled, and participants received two doses of vaccine, 1 month apart. Immunogenicity was assessed before, 2 weeks and 1 month following the second immunization. Before vaccination (baseline), 2–7% of evaluable participants had hSBA titers ⩾1:5 to any of the three indicator strains; none showed a composite response. Following 1 month after the second dose, the proportion of participants with hSBA titers ⩾1:5 were 75–100%, the proportion with a 4-fold rise was 39–99%, and the proportion with a composite response was 63% (Table 1) [US FDA, 2015b].

**Table 1.** Serum bactericidal antibody responses in subjects 11 through 24 years of age after two doses of 4CMenB administered one month apart.

	% hSBA ⩾1:5 or ⩾1:4 (95% CI)		GMT (95% CI)		% 4-fold rise (95% CI)	Composite response (95% CI)	
	Baseline	1 month post dose 2	Baseline	1 month post dose 2	1 month post dose 2	Baseline	1 month post dose 2
**V72_41 (NCT01423084): 11–17 year old adolescents from Australia and Canada (*N*=298–299)**							
**fHbp**	2 (1–4)	99 (98–100)	1.1 (1–1.1)	117 (105–130)	98 (95–99)	0	63 (57–68)
**NadA**	7 (4–10)	100 (99–100)	1.2 (1.1–1.3)	179 (163–197)	99 (98–100)		
**PorA** **P1.4**	2 (1–4)	75 (70–80)	1.1 (1–1.1)	10 (8.77–12)	39 (33–44)		
**V72_29 (NCT01214850) in 18–24 year old adults from the UK (*N*=147–193)**							
**fHbp**	69 (62–75)	100 (98–100)	11 (8.7–14)	229 (192–274)	78 (71–85)	24 (18–30)	88 (82–93)
**NadA**	60 (53–67)	100 (98–100)	6.4 (5.1–8.1)	424 (355–507)	94 (89–97)		
**PorA** **P1.4**	57 (50–64)	99 (97–100)	6.2 (4.8–7.8)	99 (80–122)	67 (58–74)		

CI = confidence interval; GMT = geometric mean titers.

* ⩾4-fold hSBA response is defined as: a post-vaccination hSBA ⩾1:16 for participants with pre-vaccination hSBA <1:4, a post-vaccination titer at least 4-fold the lower limit of quantitation (LLOQ) for participants with pre-vaccination hSBA ⩾1:4 but < LLOQ, and a post-vaccination 4-fold rise for participants with pre-vaccination hSBA ⩾ LLOQ; composite response is defined as hSBA ⩾LLOQ for all 3 indicator strains; LLOQ = 1:16 for fHbp, 1:16 for NadA for V72_41 / 1:8 for NadA for V72_29, 1:8 for PorA P1.4 for V72_41 / 1:16 for PorA P1.4 for V72_29.

Note: the table presents immunogenicity endpoints evaluated for licensure of 4CMenB in the US.

V72_29 was a phase III, randomized, controlled trial conducted in the UK among university students 18–24 years of age [ClinicalTrials.gov identifier: NCT01214850] [Read *et al.* 2014]. A total of 979 individuals were enrolled and participants received two doses of vaccine, 1 month apart. A control group received two doses of Japanese encephalitis vaccine (Ixiaro*™*, Valneva SE, Livingston, Scotland), or one dose of quadrivalent glycoconjugate meningococcal vaccine (MenACWY-CRM, Menveo*™*, GSK, Siena, Italy) followed by one dose of placebo. This was primarily a carriage study and only a subset of patients was sampled for immunogenicity assessments (*n* = 186, Table 1); of note, the study did not meet its primary endpoint as the difference in carriage between treatment groups was not significant. Before vaccination, 57–69% of evaluable participants had hSBA titers ⩾1:4 to any of the three indicator strains, and 24% showed a composite response. Following 1 month from the second dose, the proportion of participants with hSBA titers ⩾1:4 were 99–100%, the proportion with a 4-fold rise was 67–94%, and the proportion with a composite response was 88% (Table 1). The composite response was still observed in 66% of the participants 11 months after the second dose (i.e. 1 year after the start of vaccination) [US FDA, 2015b].

V102_03 was a phase II, observer-blind, randomized, controlled trial conducted in the US and Poland with participants 10–25 years of age [ClinicalTrials.gov identifier: NCT01272180] [Block *et al.* 2015]. A total of 484 participants were enrolled, and were randomized 1:1:1:1 to receive four vaccinations including two investigational vaccines for MenABCWY, 4CMenB, and a MenACWY vaccine. Prior to vaccination, 3–5% of evaluable participants had hSBA titers ⩾1:5 to any of the three indicator strains; the composite response was not measured. After 1 month from the second dose, the proportion of participants receiving 4CMenB with hSBA titers ⩾1:5 were 82–93%; the proportion with a 4-fold rise was 75–88%.

V72P10 was a phase IIb/III, observer-blind, randomized, controlled trial conducted in Chile with adolescents 11–17 years of age [ClinicalTrials.gov identifier: NCT00661713] [Santolaya *et al.* 2012]. This study was designed primarily to assess different dosing schedules of 4CMenB and its effect on immunogenicity and tolerability. A total of 1631 adolescents were enrolled, and were randomized 1:3:3:3:3 to receive placebo and four dosing schedules of 4CMenB: one dose, two doses separated by 1 or 2 months, or three doses. Before vaccination, 34–44% of participants had hSBA titers ⩾1:4 to any of the three indicator strains; after the second dose, >99% of participants had hSBA titers ⩾1:4. This study showed that a third dose of 4CMenB did not provide a clear advantage in terms of the percentage of individuals with hSBA titers ⩾1:4 and defined the two-dose schedule as the preferred schedule, with the second dose administered 1–6 months after the first.

In conclusion, these four clinical trials showed that 4CMenB induces a robust immune response against MenB test strains in adolescents following two-dose vaccine schedules.

## Overall safety profile of 4CMenB

During clinical trials, 3058 individuals aged 10–25 years received at least one dose of 4CMenB in four clinical trials (V102_03, V72P10, V72_29, V72_41) [Block *et al.* 2015; Santolaya *et al.* 2012; Read *et al.* 2014; Perrett *et al.* 2015]. Local and systemic reactogenicity data were solicited from all participants in the four studies except for study V72_29, where data were solicited from a subset of participants only. Similar solicited rates of adverse events (AEs) were reported across all studies in participants 10–25 years of age, except for severe myalgia which was reported by 12% and 13% of study participants in the V102_03 trial following first and second vaccination respectively, and by 3–7% of patients in the other three studies [US FDA, 2015b]. Severe pain following each vaccination was reported by, at most, 8% of participants in trial V72_29 [Read *et al.* 2014] compared with 29% in the V102_03 [US FDA, 2015b].

In three controlled studies (V102_03, V72P10, V72_29), where 2221 participants received 4CMenB and 2204 participants received control, nonserious unsolicited AEs that occurred within 7 days of any dose were reported by 439 (20%) 4CMenB and 197 (9%) control recipients [US FDA, 2015b]. Controls for studies V102_03 and V72P10 were saline placebo followed by a dose of MenACWY-CRM vaccine and placebo containing aluminum hydroxide; the control for V72_29 was either one dose of MenACWY-CRM vaccine followed by one dose of aluminum hydroxide placebo or two doses of Japanese encephalitis vaccine [Block *et al.* 2015; Santolaya *et al.* 2012; Read *et al.* 2014]. Unsolicited AEs that were reported among at least 2% of participants and were more frequently reported in 4CMenB recipients than in control recipients were: injection site pain, headache, injection site induration unresolved within 7 days, and nasopharyngitis [US FDA, 2015b; Block *et al.* 2015].

Across the four studies, the reactogenicity and frequency of unsolicited AEs did not seem to increase with consecutive doses of 4CMenB vaccine. [Block *et al.* 2015; Santolaya *et al.* 2012; Read *et al.* 2014; Perrett *et al.* 2015].

Overall, in these four clinical studies, 66 (2.1%) out of the 3058 participants aged 10–25 years old reported serious AEs at any time during the studies; for 5, the events were assessed as being related to 4CMenB vaccination [Santolaya *et al.* 2012; Read *et al.* 2014]. In three studies (V102_03, V72P10, V72_29), serious AEs within 30 days after any dose were reported in equal proportions in the 4CMenB and the control groups [US FDA, 2015b].

These data do not suggest safety concerns associated with 4CMenB vaccination in patients 10–25 years of age.

## 4CMenB in North America

Although meningococcal disease is a rare infection, with an incidence currently at its lowest recorded point in the US, outbreaks still arise. Between 2013–2014, MenB outbreaks occurred in two US colleges. A total of nine cases of MenB disease were reported among students or persons with links to Princeton University during March 2013–March 2014; the attack rate was calculated at 134/100,000 among undergraduates [McNamara *et al.* 2015]. Overall, four cases of MenB were recorded among undergraduate students (aged 18–22 years) attending the University of California, Santa Barbara, USA and the attack rate was calculated at 21.1/100,000 among the university population 17–22 years of age [Patel *et al.* 2014; Nolan *et al.* 2015].

In an attempt by the CDC to control the outbreak, 4CMenB was administered as a 2-dose series, used a minimum of 1 month apart [Patel *et al.* 2014; McNamara *et al.* 2015]. No other cases of MenB-related disease were reported in vaccinated students following the campaign, but one case occurred in an unvaccinated student at other university who had close contact with Princeton University students [McNamara *et al.* 2015; Watson and Turner, 2016]. Information on serious AEs was collected for 30 days after each dose from 15,346 individuals 16–65 years of age who received at least one dose. Overall, 0.3% of individuals reported serious AEs, defined according to the same criteria used in the four clinical studies as death, a life-threatening AE, hospitalizations, substantial disruption in the ability to conduct normal life functions, or a congenital anomaly/birth defect. However, only two events were considered possibly related to vaccination (rhabdomyolysis with onset 1 day after the second dose and anaphylaxis); both participants recovered.

In Canada, 4CMenB was licensed in 2013 for individuals aged from 2 months to 18 years. Thereafter, a mass vaccination campaign was implemented in the Saguenay-Lac-Saint-Jean region of Québec between May and December 2014 to control the spread of MenB disease [De Serres *et al.* 2014]. A higher incidence rate of the disease was reported for Saguenay-Lac-Saint-Jean than for the rest of Québec province since 2004 [De Serres *et al.* 2014]. The campaign targeted individuals aged 2 months to 20 years who were residing in, or attending educational institutions in the region. Occurrence of AEs was monitored by electronic questionnaire 7 days post-vaccination (active surveillance) and by using the existing local surveillance system (passive surveillance). Between May and June 2014, 43,740 individuals aged 2 months to 20 years received a first dose of 4CMenB, and the second dose was administered in September and October.

Following the campaign, the incidence of MenB IMD in the region decreased, with only two cases occurring among the 230,444 unvaccinated residents up to January 2016, and no cases reported in the vaccinated individuals [De Serres, 2016].

We focus here on safety data from adolescents and young adults only. There were 12,332 individuals who completed the electronic questionnaire between 5 May and 17 June, 2014 [De Serres *et al.* 2014]. Of these, 3250 (26%) individuals were aged between 12–20 years; 146 out of 3249 (4.5%) reported absenteeism (vaccinee or another person), while only 19 (0.6%) reported a medical consultation within 7 days post-vaccination. No vaccine-related hospitalizations were reported. The most frequently reported health problems in those individuals were general malaise (60%), local reactions (56%), gastrointestinal problems (40%) or respiratory problems (20%). Fever with onset on days 1 and 2 occurred in 6% (139/2163) of adolescents 12–16 years of age, and in 8% (83/1086) of young adults 17–20 years of age. Of the individuals reporting fever, 60% and 73%, respectively, had not measured their temperature. Antipyretic prophylaxis did not affect the probability of fever for individuals 12–20 years of age. No serious AEs were reported in individuals 12–20 years of age. The reported side effects did not dissuade vaccinees from receiving a second dose: 98% of responders aged 12–20 years stated they were likely to or definitely would receive the second dose of 4CMenB; of 146 responders who reported an AE and were 12–20 years of age, 87% intended to receive the next dose [De Serres *et al.* 2014].

With a longer follow-up period of 5 May to 11 December, 2014, 13,230 questionnaire responses were received after the first dose and 9559 were received post-second dose, for vaccinees in all age categories [De Serres *et al.* 2015]. Of these, 6% and 9% of vaccinees reported absenteeism (vaccinee or another person) or a medical consultation within 7 days post-vaccination after the first and second doses, respectively. Fever with onset on days 1 and 2 occurred in 9% of vaccinees after the first dose, and 11% of vaccines after the second dose. The most frequently reported health problems were fever, injection site reactions and general malaise. A total of two children were hospitalized due to health problems possibly related to the vaccine, including an allergic reaction to the vaccine and seizures due to high fever. Both children recovered quickly and completely. The frequency of health problems occurring within 7 days after the first and second dose of vaccine did not appear to be different to that observed within clinical trials [De Serres *et al.* 2015]. The somewhat higher rate of fever and absenteeism after the second dose might be explained by the season: the second dose was administered in September/October where a higher rate of viral infections is expected whereas the first dose was administered in May/June.

Co-administration of the 4CMenB vaccine with other adolescent vaccines at different anatomic sites, if possible, is currently recommended in the US [MacNeil *et al.* 2015], although further data are needed to clearly establish the safety and immunogenicity in case of co-administration [US FDA, 2015b].

## Why vaccinate adolescents?

### Second peak epidemiology

In the US, no vaccines for any meningococcal disease, including MenB, are recommended for healthy children younger than 10 years. The strategy for meningococcal disease is to vaccinate adolescents, because a second peak in incidence occurs in adolescents and young adults in the US. Moreover, individuals in this age group are the main carriers of *N. meningitidis* in the population [Rosenstein *et al.* 2001]. Adolescents are at high risk of getting meningococcal disease [Rosenstein *et al.* 2001]. College students as a group are at no greater overall risk than nonstudents in similar age groups; however, first-year college students living in dormitories were at higher risk for meningococcal disease [Bruce *et al.* 2001].

Importantly, the second peak in incidence reflects carriage prevalence, which peaks in 19 year olds [Christensen *et al.* 2010]. Vaccination may reduce IMD, and models suggest that MenB vaccination of adolescents alone has the potential to reduce disease in all age groups, including infants, although it would take some years for this to occur [Huels *et al.* 2014; Christensen *et al.* 2014, 2016].

Although the incidence of meningococcal disease in the US has been declining since the mid-1990s, vaccination may have had an impact on meningococcal disease in adolescents, as the ACIP recommended routine MenACWY vaccination for adolescents in 2005 [Centers for Disease Control and Prevention, 2013].

### High vaccination coverage required to evaluate impact on carriage and herd protection

Some insight on how MenB vaccination could affect disease prevalence can be gained by considering the previous introduction of MenC vaccines, although it should be noted that conjugate polysaccharide vaccines may behave differently from subcapsular protein-based vaccines. From 1999 onwards, the introduction of MenC mass vaccination campaigns followed by routine infant or toddler vaccination, in countries such as the UK, the Netherlands and Canada, dramatically reduced MenC in these countries. In the UK, disease incidence was reduced by 81% in 2 years [Trotter *et al.* 2004; Pollard, 2004]; in the Netherlands, there was a 95% decrease in the disease incidence between 2002–2012 [Bijlsma *et al.* 2014]. In Canada, there was an 83% decrease in cases of disease in 15–24 year old between 2002 and 2012 [Sadarangani *et al.* 2014].

Models indicate that vaccination against MenB might be able to have a similar magnitude of effect, even if only vaccinating in the adolescent carrier population [Huels *et al.* 2014; Christensen *et al.* 2014]. Importantly, high vaccination coverage is required to attain herd protection and to have a significant impact on carriage and disease incidence. These models were calculated based on a vaccination uptake of 85%. It must be noted that in the US, the coverage rate for the first dose of MenACWY vaccine is currently only 74–78%, and coverage rate decreases to <30% for the second dose [Elam-Evans *et al.* 2014].

In addition, the ACIP category B recommendation for the use of MenB vaccines in adolescents and young adults allows physicians to decide for each patient whether the benefits and costs of giving the MenB vaccine outweigh the risks. Taken together, this suggests that achieving a high coverage rate with MenB vaccines may be challenging among this age group.

The ability of MenB vaccines to induce herd protection is as yet unknown, and requires further investigation [Harrison, 2015]. A recent UK study estimating the effect of meningococcal vaccines on herd protection against *N. meningitidis* in university students showed that both 4CMenB and MenACWY offered carriage reduction in a subset of *Neisseria* strains 4–12 months after vaccination [Read *et al.* 2014]. However, further investigations are required as the study failed to meet its primary endpoint, aiming to demonstrate an impact on carriage at 1 month after vaccination. Ideally, a post-licensure study in a large population is required to measure the impact of MenB vaccines on public health, including the effect on herd protection [Harrison, 2015].

Of note, vaccination with 4CMenB may not be cost-effective in the US [MacNeil *et al.* 2015], regardless of the vaccination age. The main reason is the low incidence of meningococcal disease. A similar assessment was made in Canada [Hong *et al.* 2013] and European countries [Tirani *et al.* 2015; Christensen *et al.* 2016; Lecocq *et al.* 2016] and even in the UK [Christensen *et al.* 2014], where the vaccine was included in the National Immunization Program after taking into consideration opinions from various stakeholders [Wise, 2014]. The mathematical models employed so far predict that in the short term, infant immunization would have the greatest impact on public health, while in the long run, adolescent immunization would be more effective, if herd immunization is included in the model [Christensen *et al.* 2016].

### What is required for a successful MenB campaign in adolescents?

In order for MenB vaccination programs to be as successful as previous meningococcal vaccination campaigns, a public health campaign to highlight the dangers of meningococcal disease to adolescents and the wider population is required, especially in light of the unexpected university-associated MenB outbreaks in the last few years. A vaccine with a flexible immunization schedule using as few doses as possible may be helpful for effective vaccination of adolescents and young adults, as individuals in this age group are usually less compliant [Lehmann and Benson, 2009; Nelson *et al.* 2009]. To achieve high vaccination coverage required for herd protection, physicians’ support of MenB vaccination programs is indispensable. Educational programs to remind physicians of the potentially deadly, but vaccine-preventable, disease may be helpful to increase vaccination uptake. The 4CMenB vaccine might prove to play an important role in preventing meningococcal disease, as it potentially provides coverage against multiple MenB strains and has a short administration schedule.

## Conclusion

Meningococcal serogroup B (MenB) disease may pose a significant burden, especially to infants and adolescents, can be deadly, and may cause serious lifelong disabilities.

Most cases of meningococcal diseases are caused by 6 out of the 12 known serogroups: A, B, C, W, X, and Y; of these, serogroups B, C and Y are common in North America.

Meningococcal infection is generally endemic, but outbreaks can occur unpredictably.

Adolescents and young adults are the main carriers of *N. meningitidis* through the population.

In the US, the ACIP has recommended routine vaccination against serogroups A, C, W and Y for adolescents, since 2005.

There were no MenB vaccines available until 2014. There are now two vaccines available in the US.

Broad vaccination campaigns have been shown historically to have the greatest impact on disease reduction.

A high vaccination coverage is required to evaluate the impact on carriage and herd protection.
